# The Role of Genetic Resources in Breeding for Climate Change: The Case of Public Breeding Programmes in Eighteen Developing Countries

**DOI:** 10.3390/plants9091129

**Published:** 2020-08-31

**Authors:** Gea Galluzzi, Aseffa Seyoum, Michael Halewood, Isabel López Noriega, Eric W. Welch

**Affiliations:** 1 Bioversity International, Via dei Tre Denari 472/a, Maccarese (Fiumicino), 00057 Rome, Italy; m.halewood@cgiar.org (M.H.); i.lopez@cgiar.org (I.L.N.); 2 Center for Science, Technology, and Environmental Policy Studies, School of Public Affairs, Arizona State University, 411 N Central Ave, Phoenix, AZ 85004, USA; aseyoumw@yahoo.com (A.S.); EricWelch@asu.edu (E.W.W.)

**Keywords:** genetic resources, plant breeding, climate change adaptation, genebanks, policy, developing countries

## Abstract

The role of plant breeding in adapting crops to climate changes that affect food production in developing countries is recognized as extremely important and urgent, alongside other agronomic, socio-economic and policy adaptation pathways. To enhance plant breeders’ capacity to respond to climate challenges, it is acknowledged that they need to be able to access and use as much genetic diversity as they can get. Through an analysis of data from a global survey, we explore if and how public breeders in selected developing countries are responding to climate challenges through a renewed or innovative use of plant genetic resources, particularly in terms of types of material incorporated into their breeding work as well as sources of such germplasm. It also looks at the possible limitations breeders encounter in their efforts towards exploring diversity for adaptation. Breeders are clearly considering climate challenges. In general, their efforts are aimed at intensifying their breeding work on traits that they were already working on before climate change was so widely discussed. Similarly, the kinds of germplasm they use, and the sources from which they obtain it, do not appear to have changed significantly over the course of recent years. The main challenges breeders faced in accessing germplasm were linked to administrative/legal factors, particularly related to obtaining genetic resources across national borders. They also underscore technical challenges such as a lack of appropriate technologies to exploit germplasm sets such as crop wild relatives and landraces. Addressing these limitations will be crucial to fully enhance the role of public sector breeders in helping to adapt vulnerable agricultural systems to the challenges of climate change.

## 1. Introduction

The International Panel on Climate Change (IPCC) [1] defines climate change as a change in the mean value and/or variability of the climate’s properties, that persists for an extended period, usually for decades or longer. Agriculture is extremely vulnerable to climate change: increasing temperatures and declining precipitation over semi-arid regions are likely to reduce yields for a number of primary crops in the next two decades [2,3,4,5,6,7]; the intensity and distribution of pests and disease outbreaks may also become increasingly unpredictable, with serious impacts on agricultural productivity [8,9,10]. While the overall impacts of climate change on agricultural systems are expected to be negative [11], the effects may vary both in type and magnitude across geographical areas [12,13]: developing countries are likely to be most affected, not only because of their predominantly low input, rain-fed cropping systems which rely on somewhat regular weather patterns, but also because of their more rapid population growth, which determines greater pressures on agricultural production and more serious food insecurity risks [2,14,15,16].

Adaptation to climate change is the practice and process of adjusting to climate-induced adverse effects [2,14,17]. In agriculture, adaptation to climate change is about farmers’ and other stakeholders’ responses to environmental disturbances that affect their cropping systems. This may involve a broad array of alterations from livelihood and agronomic strategies to policy changes [18,19]. Among those strategies that focus on the production system itself, climate change adaptation strategies are commonly divided in two pathways: agronomic management (which tends to be short term) and genetic improvement (longer term) [9,13]. Agronomic management strategies encompass changing cultivation practices (timing or location of cropping activities, techniques of land preparation, weed/pest/disease management [18]) and the adoption of new varieties or shifting to alternative crops or crop combinations [9,13,20,21,22,23,24,25]. Genetic improvement, on the contrary, involves the development and adoption of new, better adapted varieties of the crop of interest. While farmers have always conducted genetic improvement within their informal seed networks [26,27,28,29,30], a crucial role has been played over the last century by public (and, increasingly, private) plant breeding [31,32]. Indeed, “modern”, scientific plant breeding has contributed to massive and rapid yield increases in many crops, as well as to increased tolerance to a variety of biotic and abiotic stresses; however, with more severe and frequent challenges from aggravated climate change, plant breeders are being called to place extra efforts in improving and accelerating the tools and working strategies they use, to timely provide farmers with adapted varieties [33,34,35,36], particularly in more climate vulnerable developing countries.

An essential building block for any innovation in plant breeding to occur is access to, and use of genetic diversity from existing wild or domesticated species [37,38,39,40], particularly when the genetic target dealt with is complex, as in the case of adaptive traits for climate stress responses. In developing new cultivars, breeders can start from a range of different germplasm types and germplasm sources. Among the former, landraces and crop wild relatives remain perhaps the largest reservoir of genetic diversity, including traits of tolerance or resistance to environmental stressors, even those associated with climate change [30,36,41,42,43,44,45,46,47,48,49,50,51,52,53,54]. On the other hand, advanced breeding or elite lines which have undergone pre-breeding efforts, may harbour less genetic diversity but be more “ready to use” materials for breeders, thanks to the useful information accumulated on their structure and properties [49,55,56,57,58,59]. In terms of sources, breeders can tap into the collections of national and international genebanks and germplasm research centres, which host a wide range of different materials for different crops [55,57,60], or search for the diversity they may need directly in natural or farmers’ fields, particularly in areas of origin or domestication. Recent years have witnessed the establishment of national laws and regulations that have changed the rules of germplasm access, distribution and sharing, potentially affecting the use of genetic materials by breeders [57,61,62,63,64,65]. Additional factors, including human, technical and financial resources available in any breeding programme, as well as linkages and collaborations with other national and international institutions, are likely to contribute to shaping the way breeders use genetic resources in the context of climate change adaptation [66,67].

It is against the above context that we undertook a survey of plant breeders in 19 developing countries to see how they are perceiving climate change’s impacts on their breeding objectives, and if these changes had knock-on effects on the kinds of genetic resources they use and where they access them. In particular, the objectives of the study are: (1) assessing breeders’ perception of climate change and the traits they give priority to in breeding; (2) exploring trends in the use of genetic materials in breeding programs over the course of the last two to five years (in terms of types of materials used and sources of access); (3) assessing the association between changes in the way breeders use genetic resources and their perception of climate change priorities; and (4) examining if and how regulatory, technical, financial or other issues influence the extent to which breeders are capable of innovating with genetic resources.

Section 2 presents the results of the study, Section 3 provides the discussion and conclusions; data sources, data collection procedures and methods of analysis are presented in Section 4. We chose to focus on public sector breeders only, based on the consideration that in the developing world, private breeding continues to be modest for most crops, while public support to breeding is possibly still the major avenue for the development of new varieties [66], and for focusing on traits (such as environmental stability and sustainability) or crops which may be under-researched in private sector breeding [68].

## 2. Results

Complete responses were received from 200 breeders in 18 countries, who answered in one out of three languages (English, French and Spanish), as summarized in Table 1.

Around 57% of the respondents were from national agricultural research systems, while about 24% belonged to academic institutions. Although we had targeted breeders which to the best of our knowledge belonged to public institutions, we received a few responses from the private sector, which anyhow represented only 5%. Respondents from national non-governmental or non-profit organizations totaled 6%. The remaining 8% of respondents worked in other organizations including local, community or farmers’ organizations as well as international non-governmental and non-profit organizations. Regarding gender, 146 breeders were male, while 32 were female. At the time of the survey, the average age of respondents was 49 years, and the average time they had worked as breeders was just over 17 years, of which, on average, 15 years were spent working in their current organization. Over half of the breeders (113) had a doctorate degree, while over half of the others (46) had a master’s degree.

### 2.1. Perception of Extreme Weather Patterns

**Figure 1:**
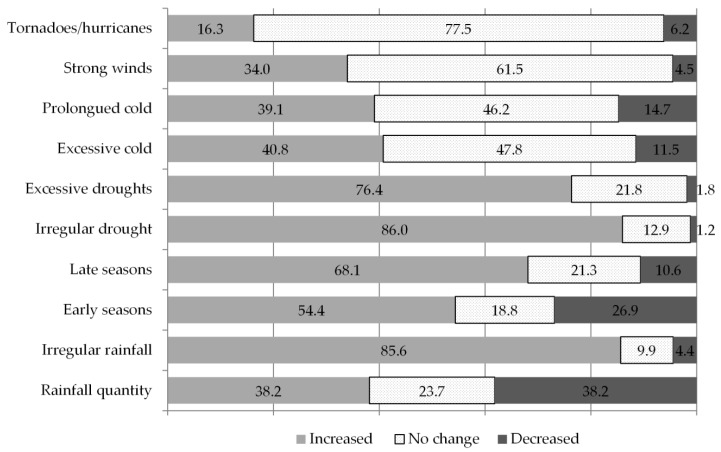
Plant breeders’ perceptions about weather patterns observed in their regions. Numbers on or next to the bars represent percentages over the total responses.

The plant breeders were asked about their perception of extreme weather events occurring in their region of work over the last five years. The results show that most breeders agreed on irregular drought periods and irregular rainfall patterns as being the most seriously increasing weather phenomena (see Figure 1). A considerable number of plant breeders also observed an increasing tendency toward a late onset of cropping seasons, a pattern that was strongly correlated to perceptions of irregular rainfall (*p*-value = 0.007864) as well as with irregular and excessive droughts (*p*-value = 0.00000002616 and 0.002548 respectively) (data not shown).

The crops which breeders work on, do not seem to significantly influence their perception of climate change. To detect if there were significant differences across regions in terms of climate change perceptions, we subdivided our responses into six geographical regions, namely Central America (with five responses), East Africa (38), East Asia (43), North Africa and the Middle East (15), South America (29), South Asia (53) and West Africa (17). Ordinal means per region revealed that the most serious problem affecting all of the areas was increasing irregularity in rainfall and drought as well as from increasing drought periods. Inter-regional differences in perceived weather patterns were not statistically significant (based on an ANOVA analyses of variance) but may provide an indication of prevailing trends. Breeders in West Africa reported to be dealing with increasing unpredictability in the onset and end of rainy and dry seasons; in East and West Africa an increase in cold weather periods was reported, while Central America was the only region where a slight decrease in overall rainfall quantity was described (in all other regions, rainfall appears to be changing little or slightly increasing). Central America also scored highest in terms of increases in strong winds and hurricanes. Late onset of seasons, more than early, was reported as an issue in many regions, namely in Central America, East Africa, East Asia, North Africa and the Middle East, and South Asia.

### 2.2. Changes in the Traits on Which Breeders Are Working

**Figure 2:**
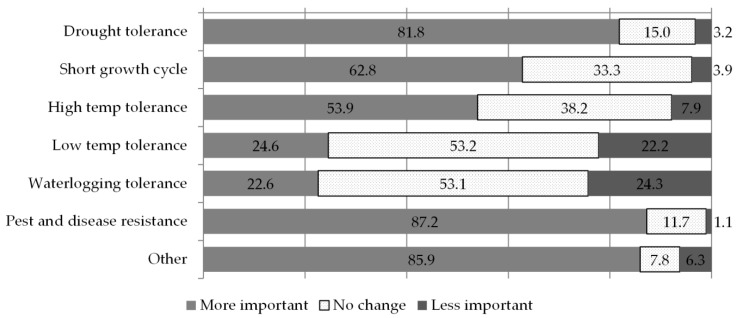
Change in the importance of traits in breeders’ work. Numbers on or next to the bars represent percentages over the total responses.

The results presented in Figure 2 describe the changes in the importance of the traits on which breeders are working. Breeding for resistance to pests and diseases is the priority that has become most important over the past five years (87%), followed closely by breeding for drought tolerance (82%), shorter growth cycles (63%) and tolerance to high temperatures (54%). In another set of questions concerning the traits that have always been most important regardless of recent changes, breeders flagged the same traits in the same order of importance. Similarly, breeding for low temperature as well as for water logging tolerance have not consistently increased in importance, and, indeed, their overall relevance even before the last five years was ranked quite low by respondents. Over 85% of breeders listed other important traits in addition to these priority ones, including quality traits (63%), traits related to tolerance to low soil fertility (20%) and yield (11%). In summary, recent years appear to have witnessed an increase in the urgency with which breeders are working on traits that already had high priority in their longer-term breeding work. The “other” traits category was chosen by a number of respondents, but 66% of these did not specify in the open text space what they meant. Those who did, flagged the importance of working for improving the quality of the crops’ final product (15% of the total “other” responses), tolerance to limiting soil conditions (acidity, salinity, low nitrogen, aluminium, 7%) and yield improvements (5%).

While our questions on changing trait importance required choosing among a list of single priority traits, the results we received reflect the complexity of breeders’ work, which entails focusing on different but somehow related traits at the same time: focusing more on pest/disease resistance and drought tolerance was related to an increase in the importance of all other breeding objectives; strong associations were also found among breeding for tolerance to low temperatures and waterlogging and between breeding for high and low temperature tolerance, as if a more encompassing breeding objective was obtaining a general improvement in temperature stress response mechanisms. The many positive associations reported between the changing importance of different traits confirm the complex, multi-trait nature of a crop’s adaptation to biotic and abiotic stresses. As expected, changes in the importance of traits during recent years showed a strong association with breeders’ perception towards changes in some of the climate patterns (Table 2): plant breeders who observe an increase in irregular and excessive drought also increase their focus towards drought resistance and shorter growth cycles; those focusing more on pest and disease resistance are those who report a more prevalent shift in seasonality and drought occurrences; those detecting excessive and prolonged cold periods (although not common among the respondents) tend to breed more for traits of tolerance to low temperatures. In summary, the recent focus of breeders has not shifted significantly from the most important traits they had been working on routinely, but it does closely mirror their perceptions of climatic events and trends.

The majority of breeders reported that farmers’ preferences for resistance to pests and diseases, drought tolerance and short-cycle varieties had increased the most, followed by high temperature tolerance; significant positive associations were detected between breeders’ increasing attention to pest/disease resistance, drought tolerance, high and low temperature tolerance and farmers’ increased preference for each of these traits. A caveat must be made here: farmers’ preferences, as described in this article, were reported by breeders in the survey and not gathered among farmers directly. We cannot therefore be certain that the breeders’ perceptions are not self-made or just intuited. In a different section of our survey we asked breeders if their institutions had specific partnerships with relevant field-level organizations that allowed them to interact with farmers and determine their needs. The majority of responses were positive; most breeders stated that their programme interacted with local, community or farmers’ organizations (over 78%) as well as with national agricultural research organizations (72%). This points to an encouraging scenario of collaboration and dialogue between researchers and grassroots/community-level organizations to ensure that breeders’ work is aligned with farmers’ needs (see Table 3).

### 2.3. Changes in the Genetic Materials Used in Breeding Programs

**Figure 3:**
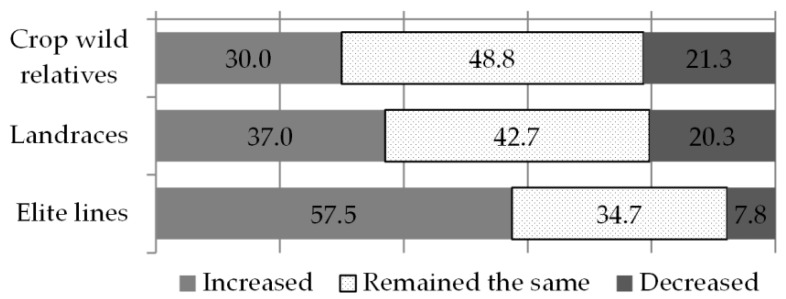
Change in the proportion of germplasm types used by breeders. Numbers on or next to the bars represent percentages over the total responses.

Having noted a positive association between breeders’ perceptions of climate change and of farmers’ needs, on the one hand, and the traits breeders’ target, on the other hand, we analysed if an association existed between breeders’ perceptions of the prevailing climate patterns and the changes in the types of genetic resources they use. First, breeders were asked to describe the relative proportion of crop wild relatives, landraces and advanced/elite lines in their routine work, and then to describe any recent (two to five years) increase or decrease of each germplasm type. Advanced or elite lines were reported as the prevalent material type in breeders’ routine work (53%), followed by landraces (25%), other kinds of materials (15%) and crop wild relatives (7%). The use of crop wild relatives showed a positive association with the use of landraces, suggesting that plant breeders who use a greater proportion of the one material type are also likely to be using more of the other. On the contrary, a trade-off between the use of elite lines and less advanced materials exists, indicating that the choice of advanced materials comes with a more likely reduction in the use of landraces and crop wild relatives (data not shown). In terms of recent change, the only category of germplasm that was reported to have increased among the majority of breeders (58%) was that of advanced/elite lines. Conversely, for landraces and crop wild relatives, the majority of responses indicated no significant change in their use (42% and 49% respectively) (see Figure 3).

The crops that breeders reported to be working on were grouped into categories: cereals and pseudo-cereals (114 responses), grain legumes (27 responses), roots and tubers (25 responses), fruits (16 responses) and other crops (18 responses). No significant differences were found between crop categories in terms of changes in the types of materials used.

A few elements of breeders’ perception of climate change were related to recent changes in the types of materials they use. The significance of these relationships is limited to landraces and elite lines since no association exists with crop wild relatives (which, as we have seen, are more scarcely used). When breeders observe increasing rainfall quantity and irregularity, or colder weather, they tend to use more landraces in their breeding work. On the other hand, those plant breeders observing later onset of seasons, tend to use smaller proportions of landraces. Elite lines are prevalent among breeders concerned with increasing drought irregularity (see Table 4).

These associations are hard to interpret or even match in literature, since all these climate challenges can potentially be met by working with both advanced materials as well as with landraces and even crop wild relatives. However, using more landraces positively correlates with a higher number of climate challenges, suggesting greater climate-related efforts or successes among breeders who explore potentially more variable reservoirs of genetic diversity.

### 2.4. Changing Sources of Breeding Materials

**Figure 4:**
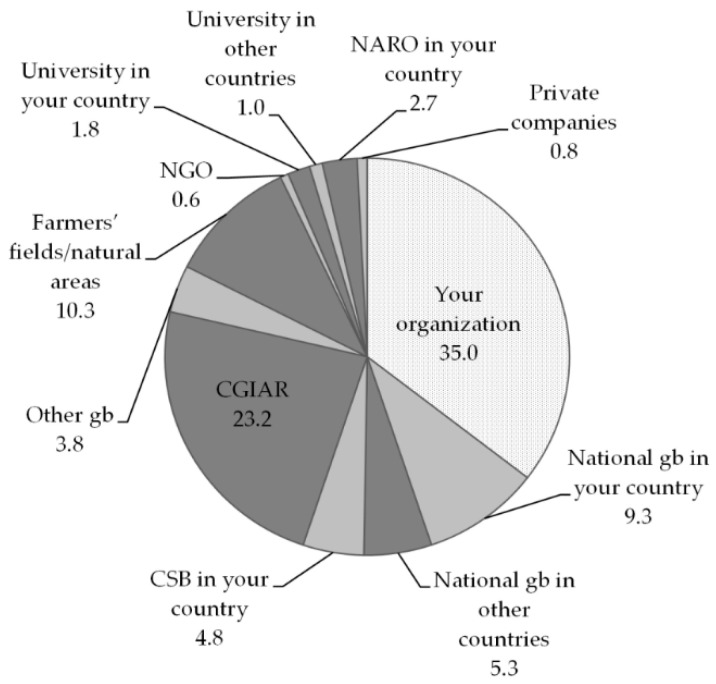
Sources of germplasm used by breeders. CGIAR, Consultative Group on International Agricultural Research; CSB, community seed bank; gb, genebank; NARO, National Agriculture Research Organization; NGO, non-governmental organization.

The majority of breeders reported to be mostly using ex situ material (82%, if we combine all internal material and that coming from genebanks and universities, excluding community genebanks). Thirty-five percent of the ex situ germplasm comes from the breeder’s own organization, and 23% comes from the CGIAR. The share of materials from the national genebank in the breeders’ own country is rather low, probably due to the fact that most respondents belonged to the NARO, which often also includes or manages the national genebank, and hence referred to this source as their “own organisation”. A less consistent, but still significant, proportion of materials also comes from farmers’ fields and natural areas (11%) and community genebanks (5%), which we consider to be a conservation strategy closer to on farm than to ex situ [69,70] (see Figure 4).

Association analyses between changes in sources of germplasm and material types highlighted a few relevant relationships. Breeders who have been using more crop wild relatives over recent years, although few in number, increasingly sourced them from a variety of ex situ collections (both public, including the CGIAR, and private, and mostly outside their countries’ borders) rather than in situ (i.e., in farmers’ or natural fields). Increasing landrace use is indeed the only trend that is associated to greater on farm sourcing; other typical sources for those breeders using more landraces are a variety of (public) institutions, all within the national territory. Increased use of elite lines was strongly associated with foreign or international sources—that is, genebanks and universities, and the CGIAR (see Table 5).

These relationships mirror what was already happening in breeders’ routine work, previous to recent years (data not shown), and are therefore not indicative of any recent change in behaviour or innovation.

### 2.5. Policy, Financial and Other Limitations

**Figure 5:**
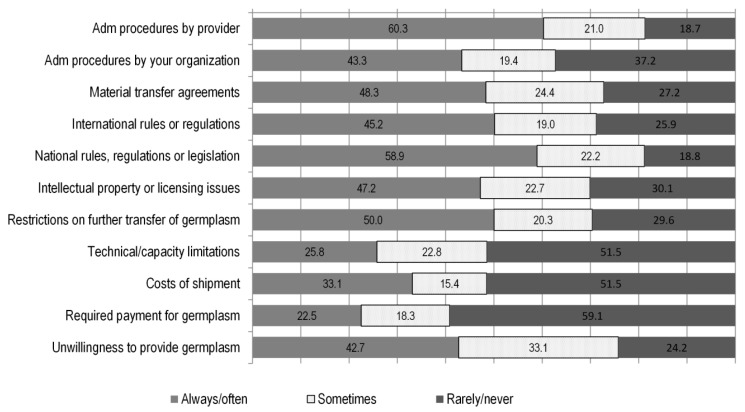
Administrative (adm)/legal and technical factors affecting breeders’ work. Numbers on or next to the bars represent percentages over the total responses.

To analyse whether other factors are related to breeders’ behaviour with respect to germplasm use, we asked breeders about if and how (always/often, sometimes, rarely/never) they are subject to limitations that affect their capacity to access and use diverse sets of germplasm. The limitations that breeders were offered to choose among were of a legal, administrative, financial and technical nature (see Figure 5). The types of restrictions that the majority of breeders reported to be always or often affected by, were of an administrative and policy nature, the most serious being burdensome procedures imposed by providers (60%) and national rules or laws (58%). These were followed by restrictions to the further transmission of the material received (50%), difficulties related to the use of material transfer agreements (48%), intellectual property regimes (47%), international rules (45%), internal administrative procedures (43%) and an unwillingness by providers to share materials (43%). Restrictions of technical (breeders’ ability to use the germplasm) and financial (payments for obtaining germplasm) nature were less frequently reported.

Some of these limitations were significantly related to breeders’ efforts to incorporate a greater proportion of specific germplasm types into their work (Table 6). Using more advanced materials, which was a widespread trend in our sample population, was related to experiencing administrative limitations both by providers and by breeders’ own organizations. The few breeders who are increasing their use of landraces and crop wild relatives experienced other limitations, including financial (for both landraces and crop wild relatives), technical capacity (crop wild relatives) and political (landraces) issues. The positive and significant association between the difficulty of using landraces and the “costs of shipment” variable is rather surprising, since respondents declared to be mostly and increasingly sourcing landraces from national or internal sources to their organization. It could be that breeders who are using more landraces are close to exhausting their “routine” sources of variation and are eager to obtain more samples of this germplasm category, which are unavailable within their closest collections. It may be that in seeking beyond these, they incur in shipping costs which they are unable to cover.

When prompted about the effect of access and benefit sharing policies on their work, most breeders reported that they were scarcely influenced by these policies (25%), although another 21% said their situation had worsened; few breeders (12%) responded that they did not know if and how these policies had affected their work. These percentages are in a way surprising if compared to the importance breeders had given to legal and administrative barriers in the previously analysed question, but may suggest that there are either other non-ABS barriers which we did not capture, or that there is not a widespread awareness and knowledge about national and international ABS frameworks and their inter-relations. Regarding in particular the effect of the International Treaty on Plant Genetic Resources for Food and Agriculture (ITPGRFA), 37% of the breeders stated that it had made little or no difference to their work, and 22% answered that they did not know. Among the surveyed countries, China (from where we received numerous responses) and Bolivia were not Parties to the International Treaty at the time of the survey (China is still not). When asked about the lack of specific technologies or tools, limited access to molecular tools and approaches (from sequencing to genomics and proteomics instruments, together with the capacity to use them) was the most urgent factor (for 68% of respondents). Infrastructure for phenotyping, controlled trials, micro-propagation and characterization followed (24%). The availability of genetic materials or information about them was deemed to be a critical limitation by only around 6% of respondents. Breeders were also asked if the budget available for their breeding programme as well as international donor funding had changed. The majority of breeders reported that both had increased (57% and 39% respectively). Positive changes in financial availability were related to a greater use of crop wild relatives in breeding, which makes sense in light of the fact that the few breeders working more with wild material had reported financial limitations.

## 3. Discussion

Overall, our results suggest that the surveyed plant breeders are well aware of increasingly urgent climate patterns in their target regions, and that their response is to increase their focus on traits that were already the highest priority in earlier years. For the vast majority of breeders, regardless of geographical or crop focus, these traits included pest and disease resistance and drought tolerance, followed by shorter growth cycles, which they believe to also be the traits most desired by farmers. Our results confirm that recent climate changes have exacerbated the breeders’ sense of urgency in addressing biotic and abiotic challenges that were already a high priority [71,72]: indeed, research has suggested that dry regions will become drier and wet regions wetter in response to global warming, a trend labelled as the “rich get richer” mechanism [73]. This tendency would naturally lead breeders to devote even more efforts to traits that have always received the highest importance in their work, such as drought tolerance, particularly in those developing countries which rely on mostly rain-fed cropping systems and are hence more heavily affected [71,74]. The prevailing tendency among the surveyed breeders was to increase their use of elite lines versus landraces and crop wild relatives. One might have expected instead to see increased reliance on more genetically diverse materials as potential sources of genetic traits adapted to changing climate changes, as highlighted in the introduction. However, increased use of landraces, albeit not widespread in our sample population, correlated with the highest number of climate challenges, suggesting that those relatively few breeders who are working more with this material type, are able to tackle specific climate change needs. However, financial, technical and policy related disincentives appear to be very influential in limiting a more widespread increase in the use of landraces, and even more so crop wild relatives. Of course, it is also possible that some of the advanced lines that breeders are increasingly using derive from introgression of traits from crop wild relatives or traditional varieties as a result of pre-breeding conducted elsewhere, thus guaranteeing the incorporation of new, potentially adaptive diversity anyway.

As far as sources of material are concerned, breeders prioritised materials coming from their own organization and secondly the international network of CGIAR Centers. A similar order of prevalence in germplasm sources has been observed in other studies. A survey among wheat breeders in developed and developing countries found that top priority was given by the majority of breeders to lines readily available within their own programmes; in developing countries, CGIAR lines and released varieties were the second most heavily used type of germplasm [75], as also emerging here. Indeed, it is widely acknowledged that the CGIAR has played a critical role in the provision of germplasm to developing countries since its inception, through its genebanks, breeding programmes and international nurseries [76,77,78,79,80]. Germplasm originally received from foreign or international programmes such as the CGIAR gradually becomes internalised into national programmes, likely making the dependence on continued international sourcing less prominent over time, compared to in-house sourcing. Indeed, international or foreign germplasm is often re-distributed by the original recipient to additional colleagues, particularly in developing countries [59]. In addition, some national programmes have consistently improved their capability of carrying out their own crosses and developing their own improved lines, with more targeted use of CGIAR improved material.

Perhaps the most interesting result of our survey is that sixty percent of the breeders confirmed that their work is always/often affected by burdensome procedures imposed by providers, and by national rules or laws regulating access and further transfer of materials. We recorded similarly high response rates with respect to the deleterious impacts of international rules and regulations, intellect property and licensing, even their own organization’s administrative procedures. Overall, this points to the lack of a supportive policy environment as a greater limiting factor than their own technical capacity to use genetic resources, their ability to pay for them or budgetary limitations. We were not clearly able to pinpoint the effect of specific international ABS rules (either those under the CBD/Nagoya or the ITPGRFA) since breeders’ responses to our questions on if/how these frameworks had affected their work (in general or with specific materials) suggested a limited effect or one of which they were not clearly aware.

Increasing the use of different material types is affected by policy and technical limitations in different ways. It is elite lines and landraces that are most affected by policy/legal issues, the former by administrative procedures imposed by providers, the latter by an unwillingness by providers to grant access to germplasm. Since breeders’ increasing use of elite lines is related to using external, foreign sources of germplasm, there may indeed be more barriers for introducing these materials, including on the phytosanitary side: it has been noted that certain countries have adopted phytosanitary policies that have led to lower acceptance rates of international genetic materials, or that some countries may not have the capacity to carry out all the analyses that their phytosanitary policies require, resulting in decreasing requests [57]. The fact that landraces are the material type specifically related to the response on providers’ “unwillingness” to share material, may be due to the long-lasting heritage of traditional and cultural knowledge and the strong sense of ownership that is associated to landraces, often instigated by civil society organizations or local and national governments [81]. Ownership and sovereignty issues become even more relevant in light of the fact that many of the breeders working with landraces are also using more materials collected from on farm sources. While on farm sourcing yields materials subject to ongoing evolutionary forces, hence potentially more adapted to climate change [57,82], it also makes it more likely to raise cultural identity and resource ownership issues. In addition to on farm sources, breeders who are using more landraces are also accessing more materials from ex situ institutions, all of which fall within the breeders’ own institution or her/his national borders. The fact that they don’t turn to the CGIAR as much, despite the centres’ facilitated access system under the conditions of the International Treaty on Plant Genetic Resources [60,83], may be due to the higher transaction costs (increased landrace use positively correlated to increased shipping costs) and possible delays due to international phytosanitary issues [57]. Furthermore, in a scenario of increasingly cumbersome international exchanges, personal contacts and closer relationships of trust may be more effective in smoothening germplasm transactions, particularly those which involve culturally-relevant materials [57].

Technical and financial challenges outweighed political/legal or ownership issues when it came respondents’ ability to access and use crop wild relatives. While there are a number of success stories around the introduction of useful traits from the highly variable pools of crop wild relatives [41,46,47,48,49,50], the transfer of alleles from wild populations tends to be slow, genetically tricky and expensive compared to when using advanced or elite lines [12,55,84]. Other limitations, such as the lack of genetic materials or information about them, only affected a minority of breeders working with wild relatives, a minority which seeks to mine an even greater variety of ex situ sources, including foreign ex situ institutions and collections from the private sector. The lack of significant in situ sourcing of crop wild relatives goes against the recommendation of using materials subject to natural climate change selective pressures and may be an issue if we also consider the far from complete CWR representation in genebanks [10,85]. However, the possibility of sourcing materials from the wild requires a knowledge of any existing in situ conservation strategies [86] as well as targeted collection missions, which many countries don’t have the finances or the technical tools to carry out. The efficiency of in situ sourcing could also be improved by greater availability and capacity to use eco-geographical and climate modelling tools, which can significantly narrow down the collection areas to be surveyed while maximising the chance to find adapted materials [87,88,89,90]. 

While most of the surveyed breeders are continuing to pursue similar strategies working with genetic resources under increasing climate change awareness, our results offer some interesting insights from the minority of breeders who are diversifying their germplasm materials and sources. Overcoming the barriers they experience may encourage a broader diffusion of those diversity-based strategies that literature describes as essential in responding to climate change [36,37,38,39,40,41,42,43,44,45,46,47,48,49,50,91]. Though some argue this potential is overstated [92,93], introducing more advanced genomic/phenotyping tools into any breeding programme has the potential to improve the power and speed of exploring large pools of diversity [94,95]. Although many molecular and genomic tools are becoming increasingly affordable, they still require substantial investment and training to be fully integrated into breeding programmes worldwide, particularly in developing countries [96,97]. The widespread diffusion of high-throughput field phenotyping infrastructure and capacities appears to be even slower [66,98,99]. The technical, technological and digital divide affecting researchers in some countries has increasingly important implications in terms of access to and use of genetic resources in breeding for climate relevant objectives. For example the opportunities to link historical climate change data with genomic analysis of germplasm stored in long-established collections, could provide an additional tool for breeders to select wild varieties with potential adaptive traits, particularly in centres of crop diversity [52]. Eco-geographical modelling tools could aid the identification of sites where to conduct multi-environment trials allowing the evaluation of germplasm across a range of climate-relevant target environments [100,101]. Financial, technology transfer and capacity building bottlenecks remain very real, particularly for certain (minor) crops and contexts that offer limited incentives for private sector involvement, for instance through public-private partnerships or consortia. Thus, increasing support from national governments and networking with foreign or international institutions will be crucial.

Above all technical trends and limitations, the persistence of high-level policy and legal bottlenecks affecting access to and use of PGRFA would deserve a more detailed and dedicated investigation than the one made possible by our survey data. Much has been written on how to smoothen national and international rules and regulations, to achieve a more mutually supportive implementation of the numerous agreements on access to and use of germplasm (and most particularly of the Convention on Biological Diversity—CBD, its Nagoya Protocol, and the International Treaty on PGRFA). It would appear that substantial work is still required, since the many actors now involved in national policy development and implementation are uncertain how to do this in practice. This scenario risks negatively affecting researchers and crop breeders, who instead could and should be playing an important role, hand in hand with farmers, in adapting agriculture to future climate challenges.

## 4. Materials and Methods

Data for this study were collected in 2013 through a web-based survey designed by a research team from the Centre for Science, Technology and Environment Policy Studies of Arizona State University and Bioversity International. The study targets were plant breeders in national programmes dealing with food security crops in their countries. Target countries were 19 developing countries across Asia, Africa and Latin America (namely Bhutan, Bolivia, Brazil, Burkina Faso, China, Costa Rica, Ethiopia, Guatemala, India, Ivory Coast, Jordan, Kenya, Morocco, Nepal, Peru, Philippines, Rwanda, Uganda and Zambia). Many of these are countries where Bioversity International has been running projects and has contacts within the plant genetic resources community. In addition, the selection also took into account countries’ human development index and their climate vulnerability.

The sample frame for the target population was prepared by collecting contacts of potential respondents from electronic sources, including country reports from the Global Partnership Initiative for Plant Breeding Capacity Building, the African Crop Science Society and a list of national and international workshop participants, and through Bioversity International’s network of national project partners. Complete contact information for approximately 1092 potential respondents was collected within the 19 countries. The team checked for repetition of names or incorrect email addresses (for example, through pinging) before administering the survey. The survey was pre-tested by the study team members. The final version was translated to English, French and Spanish, written into the Sawtooth software© and administered online. Potential respondents received an email describing the project and requesting their participation in the survey, with an appropriate consent statement. The letter provided information about the survey and its purposes as well as the link to the instrument, a unique identification and an individual password. The survey contained 65 questions covering a wide range of issues (technical, policy, financial) regarding breeders’ work; the questions we are analysing in this paper focused on breeders’ perceptions of climate change and of farmers’ climate-related needs over the past two-to-five years, the priority traits they work on, the types of germplasm they use and the sources of access for such germplasm. For priority traits, types and sources of germplasm, breeders were asked both about their business-as-usual behaviour and about any changes occurring over the last two-to-five years. Other questions analysed here concerned the institution breeders worked in, the resource allocation for their programme, as well as gender, age, academic qualifications and breeding experience. Except for age and years of work as breeders, all the other questions were multiple choice, with several pre-defined responses. Finally, we also looked into breeders’ responses to an open ended question on what tools they would need to improve their breeding work and efficiency. All categorical responses were coded numerically for subsequent analyses, which employed basic descriptive statistics such as mean, frequency and association analyses. All data analysis was performed in R [102].

## Figures and Tables

**Table 1:** Sample frame of the survey.

Language	Country	Respondents
English	China	38
	Ethiopia	15
	India	33
	Jordan	6
	Kenya	5
	Nepal	20
	Philippines	5
	Rwanda	5
	Uganda	12
	Zambia	1
French	Burkina Faso	7
	Cote d’Ivoire	10
	Morocco	9
Spanish	Bolivia	3
	Brazil	12
	Costa Rica	3
	Guatemala	2
	Peru	14
Total		200

**Table 2:** Correlation between the change in importance of traits in breeding and perceived changes in climate.

		Change in Breeders’ Priority Traits					
		Drought Tolerance	Short Growth Cycle	High Temp Tolerance	Low Temp Tolerance	Waterlogging Tolerance	Pest/Disease Resistance
Perceived changes in climate	Rainfall quantity	−0.07	−0.11	−0.02	0.05	0.03	0.01
	Irregular rainfall	0.11	0.12	0.03	−0.03	−0.09	0.11
	Early seasons	−0.08	0.07	0.11	0.10	−0.12	0.13
	Late seasons	0.12	0.11	0.14 *	−0.16	−0.05	0.23 ***
	Irregular drought	0.18 **	0.24 ***	0.10	−0.05	−0.06	0.15 *
	Excessive drought	0.20 **	0.08	0.08	0.06	−0.02	0.18 **
	Excessive cold	0.13	0.04	0.11	0.31 ***	0.15	0.08
	Prolonged cold	0.03	0.03	0.02	0.27 ***	0.07	0.10
	Strong winds	−0.06	0.03	−0.05	0.05	−0.06	0.03
	Tornadoes/hurricanes	0.03	0.03	-0.01	0.01	0.02	−0.02

* Significant at the 0.1 probability level. ** Significant at the 0.05 probability level. *** Significant at the 0.01 probability level.

**Table 3:** Association between breeders’ priorities and breeders’ perceived farmers’ preferences for traits.

		Change in Breeders’ Priority Traits					
		Drought Tolerance	Short Growth Cycle	High Temp Tolerance	Low Temp Tolerance	Waterlogging Tolerance	Pest/Disease Resistance
Change in famers’ priority traits	Drought tolerance	0.35 ***	0.15 **	0.11	−0.10	−0.05	−0.01
	Short growth cycle	0.22 ***	0.53 ***	0.08	0.04	−0.08	0.09
	High temp tolerance	−0.06	0.09	0.57 ***	−0.04	−0.02	0.17 **
	Low temp tolerance	−0.02	−0.08	−0.07	0.47 ***	−0.02	0.06
	Waterlogging tolerance	−0.04	−0.07	−0.08	0.02	0.41 ***	0.02
	Pest and disease resistance	0.01	0.09	0.03	0.13 *	0.11	0.20 ***

* Significant at the 0.1 probability level. ** Significant at the 0.05 probability level. *** Significant at the 0.01 probability level.

**Table 4:** Association between breeders’ perception of climate change and changes in their use of germplasm.

	Crop Wild Relatives	Landraces	Advanced/Elite Lines
Rainfall quantity	0.12	0.15 *	−0.06
Irregular distribution of rainfall	−0.01	0.15 **	−0.02
Early seasons	−0.02	0.04	−0.19 **
Late seasons	−0.09	−0.17 **	0.09
Irregular drought	−0.08	−0.01	0.22 ***
Excessive droughts	−0.00	0.07	0.07
Excessive cold	0.01	0.22 ***	−0.09
Prolonged cold	−0.02	0.22 ***	−0.08
Strong winds	0.09	0.01	−0.12
Tornadoes/hurricanes	0.02	0.10	−0.01

* Significant at the 0.1 probability level. ** Significant at the 0.05 probability level. *** Significant at the 0.01 probability level.

**Table 5:** Association between changing material types and sources of breeding material.

		Germplasm Types		
		Crop Wild Relatives	Landraces	Advanced/Elite Lines
Sources of genetic materials	Your own collection or the collection/genebank in your organization	0.10	0.19 **	0.03
	Farmers’ fields or natural areas	0.09	0.28 ***	−0.07
	National genebanks in your country	0.13	0.29 ***	−0.03
	National genebanks in other countries	0.28 ***	0.03	0.17 *
	Farmer community genebanks in your country	−0.06	0.32 ***	0.08
	CGIAR	0.19 *	0.04	0.23 **
	Other genebanks	0.05	−0.03	0.13
	Non-governmental organizations	0.23 *	−0.09	0.05
	University researchers in your country	0.15	0.11	0.04
	University researchers in other countries	0.08	−0.01	0.23 **
	Researchers in national agricultural research organizations	0.14	0.22 **	0.12
	Private companies	0.25 **	−0.05	0.17

* Significant at the 0.1 probability level. ** Significant at the 0.05 probability level. *** Significant at the 0.01 probability level.

**Table 6:** Association between limiting factors and changes in the use of germplasm types.

		Germplasm Types		
		Crop Wild Relatives	Landraces	Elite Lines
Limiting factors	Administrative procedures set by providers	0.03	0.10	−0.13 *
	Administrative procedures set by your organization	0.07	−0.01	−0.14 *
	Material transfer agreements	−0.01	−0.01	−0.02
	International rules or regulations	0.05	0.03	−0.08
	National rules, regulations or legislation	−0.04	−0.02	−0.09
	Intellectual property or licensing issues	−0.08	−0.11	−0.06
	Restrictions on the further transfer of the germplasm to third parties	−0.05	0.09	−0.00
	Technical/capacity limitations	−0.23 ***	−0.00	−0.10
	Costs of shipment	−0.18 **	0.22 ***	−0.02
	Required payment for germplasm	−0.14	0.12	−0.03
	Unwillingness of other organizations to provide germplasm	−0.10	−0.18 **	0.04

* Significant at the 0.1 probability level. ** Significant at the 0.05 probability level. *** Significant at the 0.01 probability level.
